# Spatial Variation in Contraceptive Practice Across the Districts of India, 1998–2016

**DOI:** 10.1007/s40980-021-00092-9

**Published:** 2021-08-19

**Authors:** Shareen Joshi, Uttamacharya, Kakoli Borkotoky, Abhishek Gautam, Nitin Datta, Pranita Achyut, Priya Nanda, Ravi Verma

**Affiliations:** 1 Associate Professor of International Development, Edmund Walsh School of Foreign Service, Georgetown University, 3700 “O” St. NW, Washington, DC 20057 USA; 2 Formerly With International Center for Research On Women, Senior Manager, MLE, Population Services International, New Delhi, India; 3 International Center for Research On Women, Asia Regional Office, New Delhi, India; 4 Senior Program Officer, Bill & Melinda Gates Foundation, New Delhi, India

**Keywords:** India, Fertility, Contraception, Fertility Transition, Spatial analysis

## Abstract

India is currently one of the most demographically diverse regions of the world. Fertility and mortality rates are known to show considerable variation at the level of regions, states and districts. Little is known however, about the spatial variations of the contraceptive usage—a critical variable that is relevant to fertility as well as health policy. This paper uses data from four national population-based household surveys conducted between 1998 and 2016 to explore district-level variations in the contraceptive prevalence rate. We find no clear evidence of convergence. The gap between the best and worst performing districts is more than 70 percent across the four rounds and does not diminish over time. We also find considerable evidence of spatial clustering across districts. Districts with high prevalence concentrate in Southern states and more recently, in the Northeast of the country. Our analysis suggests that female literacy and health care infrastructure are important correlates of spatial clusters. This suggests that investments in women’s human capital and health-care infrastructure play a role in expanding women’s opportunities to time their births.

## Introduction

India, home to approximately a fifth of the world’s population, is currently one of the most demographically diverse regions of the world. Though its total fertility rate (TFR) has halved since 1980 and is now close to replacement level, the pace of this decline has varied significantly across its regions (Cassen, 2016; Drèze & Sen, 2013; Guilmoto, 2016; Haub & Sharma, 2015; Munshi et al., 2016).^1^

Spatial variations in India’s demographic outcomes are known to be driven by a complex set of economic, political, social and cultural factors. Health infrastructure, the supply of contraception and policies also play a role.^2^ India was one of the first countries in the world to establish a national population program (Harkavy & Roy, 2007; Solinger & Nakachi, 2016). There was however, considerable variation in policies across states. Tamil Nadu and Andhra Pradesh for example, two states that experienced rapid fertility decline, established strong maternal and child health systems at the grassroots level that provided women not only contraceptive services, but maternal and child health services more generally (Drèze & Sen, 2002, 2013; Kishor, 1994). Other states relied heavily on permanent methods of contraception that required almost no investment in long-term health infrastructure (Harkavy & Roy, 2007; Solinger & Nakachi, 2016). Though it is difficult to make causal inference from these studies, there appears to be a clear association between investment in population policies and fertility decline in certain regions (Cassen, 2016; Government of India, 2017; Jeffery & Basu, 1996).

While the academic debates on the effectiveness of family planning is unresolved, there has been a growing consensus on the need to expand voluntary modern contraceptive options in India.^3^ The most recent estimate of the national contraceptive prevalence rate (CPR) suggests that 53.3 percent of Indian women rely on any methods (World Bank, 2018). This estimate conceals considerable state-level variation. The gap between the best and worst performing states however, is more than 55 percentage points (New et al., 2017). There is also some evidence that supply has stagnated in the past decade, and nine states have experienced a decline in fertility without a corresponding increase in utilization of modern contraception (IIPS, 2017). Most of this analysis however, uses data that is ill-suited for analyzing national and state-level trends—changes in sampling frames, survey protocols and state-level implementation strategies has been argued to generate puzzling trends (Desai, 2018).

To date, there has been no published analysis of contraceptive prevalence at the level of Indian districts. This paper attempts to fill this gap in the literature by examining the spatial variations of contraceptive use in India at district level using national population-based household surveys that span the past 20 years. We focus on 515 districts and survey population that is common to all four surveys (Dandona, Pandey, & Dandona, 2016).

We focus specifically on the variations in the contraceptive prevalence rate (CPR), and modern contraceptive prevalence rate (MCPR). We begin by examining whether the districts of India have shown convergence in the past twenty years. Convergence—a reduction in inequality across distinct geographic regions as lagging areas catch up with those that surged early on—has been investigated for a number of demographic and health indicators at the cross-country and cross-state level (Dorius, 2008; Goli and Arokiasamy, 2014). To explore this, we first look at aggregate trends and note the highest and lowest values of these variables across states and districts over time. Next, we look at more localised forms of convergence by examining patterns of spatial clustering at the district level. We compute Moran’s I Index to measure the extent of autocorrelation among the neighbouring districts within survey rounds and also use Local Indicators of Spatial Association (LISA) to observe spatial autocorrelation at the local level. In the final step, we use spatial regression models to explore the determinants of CPR and mCPR, with further tests of, and adjustments for, spatial correlations.

Our results find no overall evidence of convergence in the CPR across the districts of India. We find significant spatial clustering in the CPR as well as the mCPR—some districts have experienced significant change over the past 20 years, while others have remained largely stagnant and these districts cluster together on maps of the country. Since these changes are being documented *within* states and within single rounds of the survey, we do not believe that these can be entirely driven by the issues of survey design and implementation. We find that the CPR appears to be correlated with factors that are well-known in the literature on demographic change: female literacy and the availability of health care infrastructure. The results are robust to different models and specifications. In other words, the evidence suggests that *local* investments in human capital as well as human capital shape women’s opportunities to adopt contraception and control their fertility in India. Long-term convergence in contraceptives will thus depend on the convergence of such investments at the local level.

These results should be of considerable interest to academics as well as policy-makers as they show the challenges India faces in meeting its commitment to the goal of universal access to modern contraceptives by 2030. Policies need to be carefully targeted to specific districts and had to consider the underlying situation of low levels of female literacy, female employment and access to health care as these vary enormously over the regions, states and districts of India.^4^

## Methods

### Data Sources

This study draws on national population-based household surveys that have been used to measure national and sub-national health outcomes in India for the past 20 years. We combine data from three rounds of District Level Household Survey (DLHS-1:1998–99, DLHS-2:2002–04, DLHS-3:2007–08) and National Family Health Survey (NFHS-4: 2015–16) to generate the district level estimates. Additionally, we use Census data for 2001 and 2011 to extract district-level values of population density, average household size, sex ratio and female work participation. This study excluded the first three rounds of the NFHS because of the small sample size and absence of district-level identifiers.^5^ The four rounds of survey data used in this study are representative at the national-level and used similar survey tools to collect information.

Comparing data across rounds however, presents many challenges. First, is the challenge of sampling frames. Two states (Nagaland & Tripura) were not surveyed in the DLHS-2 survey. The DLHS 2 and 3 were conducted with the 2001 census as the sampling frame, while the NFHS-4 used the 2011 census. The creation of new states and districts over the study period led to several changes in the survey implementation. Appendix Table A1 highlights the scale of this problem. We address the issue by keeping only the states that were covered in all four of the surveys and using the states and districts from DLHS-1 as the reference (see Table A1).^6^ Matching new districts to the old “parent” districts in previous rounds of the survey however, does not fully address the challenge. New districts largely emerged in underdeveloped states such as Bihar, Madhya Pradesh and Uttar Pradesh (Table A1). In a sample of districts where new districts are renamed to the old parent districts, population-level averages are still unlikely to be a reliable measure to construct or interpret a trend.^7^ Since we are unable to address this challenge, our analysis focusses as much as possible on cross-sectional analysis rather than the construction or interpretation of trends.

Second, there is the challenge of respondent selection. The surveys differed in inclusion criterion for respondents based on their age and marital status (Dandona et al., 2016). Therefore, this study focused on currently married women aged 15 to 44 since this age group was common in all the surveys.

Third, there is the challenge of major differences in survey design and implementation. All rounds vary in the number of respondents, the length of the questionnaire, the protocols about privacy, and the implementing partners. These concerns are the largest for the fourth and most recent round. This sample was five times bigger than the first round, and it featured triple the number of questions. The larger sample size and complexity of the survey resulted in many changes in the criteria of selection of field organizations and implementation strategies at the state-level. The level of supervision provided by the International Institute for Population Sciences (IIPS) was vastly different than the previous three rounds. Moreover, the mode of data collection has changed from paper and pencil interviewing to computer assisted personal interviewing (CAPI). This may have changed the type of interviewers, the protocols followed and the responsiveness of respondents to sensitive questions. To minimise the challenges of comparing data across rounds, we rely as much as possible on the women’s questionnaire and the simplest questions that were consistently asked across rounds, and were asked in considerable detail. The questions on education, fertility and contraception featured several follow-up questions and for each of these questions, there was cross-validation in a separate section of the survey. We also believe that these issues are more likely to be problematic for analysis at the state- and national-level, rather than the district level.

To summarize, our working sample includes aggregates for women aged 15–44 across 515 districts that are drawn from the DLHS-1. We further drop the two states that were not surveyed in the DLHS-2. Our final sample consists of 490 districts of India.

### Study Variables

The outcome variables of interest are the CPR and mCPR. Given that India is one of the most demographically diverse regions in the world, with states that are at different stages of demographic transition, we include a broad mix of explanatory variables that are known to affect fertility transitions (Bongaarts, 2001; Bongaarts & Potter, 2013). The first set of variables is socio-economic. In our bivariate explorations of contraceptive prevalence, we rely heavily on a measure of female literacy as an explanatory variable. In multivariate regressions, we also include the female labor force participation which includes participation in the formal as well as informal sector. Though this may be chosen simultaneously as contraception, we include it to capture the opportunity costs of women’s time as well as their access to information and health services. Though the estimates from such regressions cannot be interpreted causally, robust associations between these variables are of interest in the literature of the role of female human capital in contraceptive adoption and fertility decline (Schultz, 2008). We also include controls for population density and the fraction of a district that belongs to officially recognized disadvantaged communities, notably Scheduled Caste (SC) and Scheduled Tribe (ST) groups as well as Muslims.^8^ These variables are known to be correlated with human capital outcomes in India (Anderson et al., 2018; Government of India, 2017; Joshi, Kochhar, & Rao, 2017). We combine the SC and ST groups together and refer to them as SC/ST for the remainder of this paper.

Our next set of variables is demographic. Here we include the district-level sex-ratio, the mean age at marriage and the average size of households. Previous research has shown that a sex-ratio skewed towards males, a lower age at marriage and the practice of patrilocal exogamy into intergenerational extended households are all associated with higher levels of gender inequality and thus to lower female bargaining power in the adoption of modern contraception (Clark, 2000; Dreze & Murthi, 2001; Dyson & Moore, 1983; Jayachandran, 2017; Rahman & Rao, 2004).

Finally, our list of explanatory variables includes the fraction of the district population that reports a completion of 3 ante-natal care visits in a six-month period after the birth of a baby (defined over households that reported a birth in the past year). Summary statistics of all key variables, for all rounds of the survey, are presented in Table 1.

**Table 1:** Summary statistics for the key variables used in analysis

DLHS-1 (1998–99)	N	Mean	Std. Dev	Min	Max	Moran’s I
Urban Population	490	20.61	17.21	0.00	100.00	0.242
Female literacy	490	43.29	19.41	10.01	98.09	0.640
Age at marriage (for women)	490	17.35	1.48	14.37	21.85	0.777
ANC utilization	490	60.06	26.64	0.00	100.00	0.718
Scheduled caste/tribe	490	30.82	20.16	1.84	99.19	0.535
Muslim	490	11.53	15.83	0	99.10	0.689
Women received3 + ANC	490	39.06	27.49	0	100	0.777
Population Density	490	674.85	2053.88	2.40	24,820.83	0.097
Sex ratio	490	936.11	62.48	591.27	1147.03	0.699
Female labor force participation	490	32.45	9.60	7.44	55.15	0.606
Average household size	490	5.42	0.780	3.69	8.36	0.830
DLHS-2 (2002–04)						
Urban Population	490	31.43	14.03	0.00	100.00	0.117
Female literacy	490	52.13	18.02	13.38	99.67	0.687
Age at marriage (for women)	490	17.81	1.51	15.19	22.37	0.779
ANC utilization	490	76.23	21.25	22.06	100.00	0.689
Scheduled caste/tribe	490	31.16	19.00	0.06	99.04	0.530
Muslim	490	11.86	15.85	0	99.62	0.685
Women received3 + ANC	490	52.86	26.67	8.82	100.00	0.809
Population Density	490	674.85	2053.88	2.40	24,820.83	0.097
Sex ratio	490	936.11	62.48	591.26	1147.03	0.699
Female labor force participation	490	32.45	9.60	7.44	55.15	0.606
Average household size	490	32.45	9.60	7.44	55.15	0.830
DLHS-3 (2007–08)						
Urban Population	490	22.11	17.97	0.00	100.00	0.252
Female literacy	490	57.52	19.52	13.33	99.82	0.738
Age at marriage (for women)	490	17.70	1.87	13.30	22.82	0.815
ANC utilization	490	77.55	18.56	29.12	100.00	0.678
Scheduled caste/tribe	490	35.11	21.45	1.48	99.72	0.630
Muslim	490	11.47	16.45	0.00	99.55	0.687
Women received3 + ANC	490	55.00	26.81	7.65	100.00	0.827
Population Density	490	759.83	2148.98	2.00	26,553	0.118
Sex ratio	490	945.26	62.38	533.57	1184.40	0.599
Female labor force participation	490	31.68	9.02	10.44	52.23	0.574
Average household size	490	4.96	0.71	3.42	7.64	0.779
NFHS-4 (2015–16)						
Urban Population	490	27.90	20.32	0.00	100.00	0.339
Female literacy	490	69.87	16.44	23.59	100.00	0.712
Age at marriage (for women)	490	18.38	1.56	14.55	23.10	0.743
ANC utilization	490	85.51	14.51	8.68	100.00	0.651
Scheduled Caste/Tribe	490	35.69	20.45	0.48	99.78	0.547
Muslim	490	11.89	16.40	0	99.88	0.694
Women received3 + ANC	490	68.45	21.86	3.79	100	0.756
Population Density	490	759.83	2148.98	2	26,553	0.118
Sex ratio	490	945.26	62.38	533.57	1184.40	0.599
Female labor force participation	490	31.68	9.02	10.44	52.23	0.574
Average household size	490	4.96	0.71	3.42	7.64	0.779

### Data Analysis

We first examine the aggregate patterns in our variables at the national and sub-national level. We rely on ArcGIS to generate descriptive maps and GeoDa to construct spatial models. Chloropleth maps for CPR and mCPR for the four time periods (1998–99, 2002–04, 2007–08, 2015–16) provide an initial insight into the extent of clustering in the data.

Univariate and bivariate Moran’s I Index is calculated to check for spatial autocorrelation at district level. Moran’s I estimations helps measure the degree to which data points are similar or dissimilar to their spatial neighbors. For any two neighbors, $i$ and $j$ Moran’s I is calculated as:

$$ Moran^{\prime}s I = \frac{n}{S} \times \frac{{\mathop \sum \nolimits_{i = 1}^{n} \mathop \sum \nolimits_{j = 1}^{n} w_{ij} z_{i} z_{j} }}{{\mathop \sum \nolimits_{i = 1}^{n} z_{i}^{2} }} $$

$z_{i}$: standardized variable of interest, i.e. the difference between an observed observation and the mean;

S: the sum of all the spatial weights, i.e. $S = \mathop \sum \nolimits_{i = 1}^{n} \mathop \sum \nolimits_{j = 1}^{n} w_{ij}$;

$w_{ij}$: spatial weight matrix;

$n$: total number of districts.

Moran’s I requires a spatial weights matrix to address spatial correlation of the outcome variable with the exploratory variables (Anselin et al., 2006).^9^ Given the large sizes of many Indian districts and the multiplicity of population centers within these districts, we used the queen’s criterion for contiguity based spatial weights available in GeoDa for this analysis.^10^ We estimate the significance of Moran’s I using the method of randomization with 999 permutations, with an associated pseudo p-value of 0.05, 0.01 and 0.001. We check the sensitivity of the obtained result to different randomizations and test the null hypothesis of random spatial processes, but report only the results of the 0.001 pseudo p-values (Anselin et al., 2006; Anselin et al., 2019).

Moran’s I can be interpreted to provide an overall estimate of spatial autocorrelation in the data. Positive (negative) values indicate positive (negative) spatial autocorrelation. Values of I range from − 1 (indicating perfect dispersion) to + 1 (perfect correlation). A zero value indicates random spatial pattern. Estimates of z-values, obtained from randomization are used to test the null hypothesis that the spatial processes promoting the observed pattern of values in the CPR and mCPR is random chance. This approach can thus reject the null hypothesis of spatial randomness in favor of an alternative of clustering.

While Moran’s I can provide insights on the existence of clustering, it does not provide any indication of the location of the clusters. Next, we use the univariate LISA to measure the correlation of neighbourhood values around a specific spatial location. This provides insights into the localized levels of clustering in the data. We also use the bivariate LISA model to explore the possible correlates of this clustering. The bivariate LISA measures the local correlation between a variable and the weighted average of another variable in the neighbourhood. Estimating the bivariate LISA requires taking the cross-product of the standardized values of one variable at location (e.g. CPR) with those of the average neighbouring values of another variable (e.g. female literacy). We report these results in the form of cluster maps with a significance level of 5%. Significance levels of 1% and 0.1% and the Bonferroni bound procedure was also used to check the robustness of the findings (though we do not ultimately present these results). We present the results that best represent the important High-High and Low-Low clusters in each round of the survey.

Finally, we use spatial regression models to examine the role of the full set of explanatory variables on our set of dependent variables. We rely on spatial regression tools in GeoDa as well as STATA 16.^11^ We start with a basic OLS specification of our outcome variables with a set of explanatory variables. Diagnostic tests for spatial dependence are conducted and serve as a guide for refining the model to incorporate spatial dependence.

To address the issue of spatial clustering, we consider two types of models to adjust for spatial dependence: a spatial lag model and a spatial error model.^12^ The spatial lag model is as follows:

$$y = \alpha + \rho Wy + \beta X + \varepsilon;$$

Here y is outcome variable; $\alpha$ is an intercept, $\rho$ is the spatial lag parameter, $W$ is the spatial weight matrix, β is the vector of regression parameters, $X$ is the matrix of exogenous explanatory variables. It is assumed here that the error term ε is identically and independently distributed (iid) (Anselin et al., 2006). Note that in this model, spatial dependence is driven by the clustering of the observable variable. OLS estimates in this instance are biased and inconsistent (Anselin et al. 2006).

The alternative spatial model we consider is the spatial error model. This assumes that the pattern of spatial dependence is attributable to unmeasured covariates that are orthogonal to the included regressors. The model is as follows:

$$ y = \alpha + \rho Wy + \beta X + \varepsilon $$

with $$\varepsilon = \lambda W\varepsilon + \zeta$$.

where, $\lambda$ is the spatial autoregressive parameter$.$ Error $\zeta$ are iid, and W is spatial weight. This is a special case of regression with a non-spherical error term and in which OLS, although unbiased, is inefficient. Therefore, here, the spatial regression considers proximity among geographical units through the weight matrix W.

Both spatial error and spatial lag models are estimated by maximizing the corresponding likelihood functions (Anselin et al., 2006). A large literature on spatial modeling compares these models and demonstrates that it is quite common to reject the null of spatial dependence in both models, making it difficult to ascertain the true source of spatial clustering (see for example, Baller et al., 2001, Gibbons & Overman, 2012, LeSage, 2015). More complex models such as the General Nesting Spatial Model (GNS) are able to distinguish between the two type of spatial dependence, but are generally weakly identified and thus of limited practical use (Gibbons & Overman, 2012, LeSage 2015). We present both models in this paper. We believe however, that in the future, if the reliability of population surveys of India can be improved, panel models applied to district data could provide useful insights into the specific sources of spatial clustering.

## Results

### Broad Trends

**Fig. 1:**
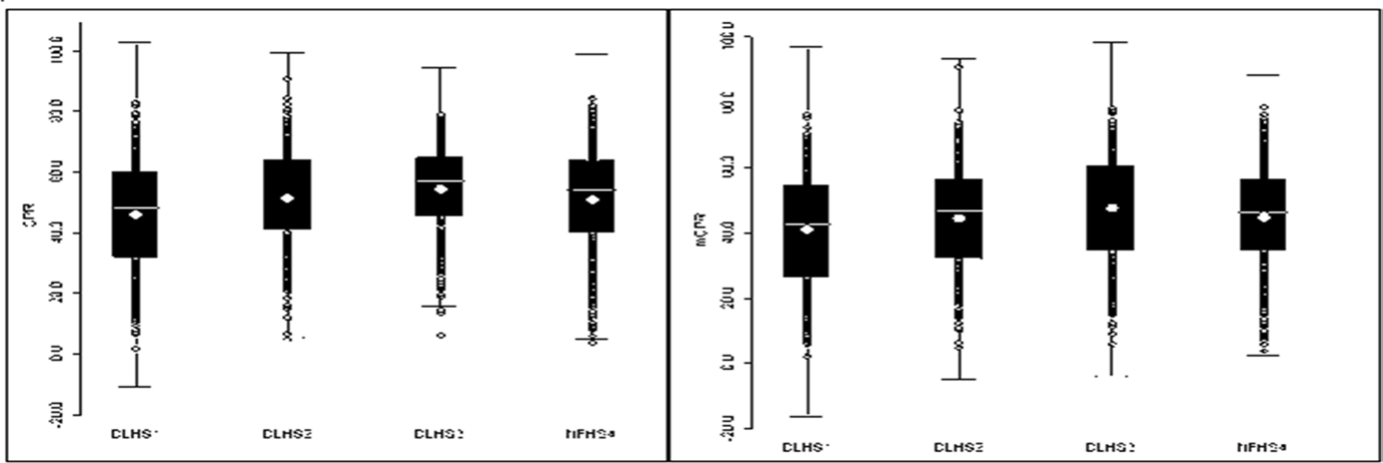
Box Plot summarizing district level CPR and mCPR for DLHS-1 (1998–99), DLHS-2 (2002–04), DLHS-3 (2007–08), NFHS-4 (2015–16)

Figure 1 presents box plots of district level estimates of the CPR and mCPR for the four time periods. The range of variation across Indian districts is quite striking. In all rounds of data, the minimum observed prevalence of the CPR and mCPR, averaged at the district-level, was below 7 percent, and the maximum was 80—90 percent. Previous analysis, conducted at the state level, reported a gap between the best and worst performing states of approximately 55 percentage points (New et al., 2017).

The box-plots also show that median CPR and mCPR increased until the most recent sample period. The CPR went from around 45% in 1998–99 to around 58% in 2007–08 and then declined to around 50% in 2015–16. The mCPR follows a similar pattern. The decline in the most recent survey has been widely noted among researchers and in media (Desai, 2018; Rai, 2017). We also note that the interquartile range has decreased between 1998–99 and 2007–08. At first glance, this is evidence of convergence of CPRs across districts—the gap between districts declined over this sample period. These estimates however, must be interpreted cautiously. As discussed earlier, changes in survey methodology and sampling frames across rounds could drive these results. In the most recent round, the increase in the sample population, the increased length of the questionnaire, and decreased level of oversight from the IIPS may have led many enumerators to skip sections of the questionnaire that required privacy, thus contributing to lower levels of contraceptive prevalence than in previous rounds. (Desai, 2018). Moreover, the change in the sampling frame and the creation of new states and new districts can reduce the reliability of the estimates. Even though we matched the new districts to their original districts, any population-level average is unlikely to be a reliable measure to construct or interpret a trend.

Given these data limitations, we draw no clear conclusions on convergence at the national level. We turn instead to the analysis of spatial clustering to examine the patterns at the district level over the four rounds of our surveys.

### Spatial Models

**Fig. 2:**
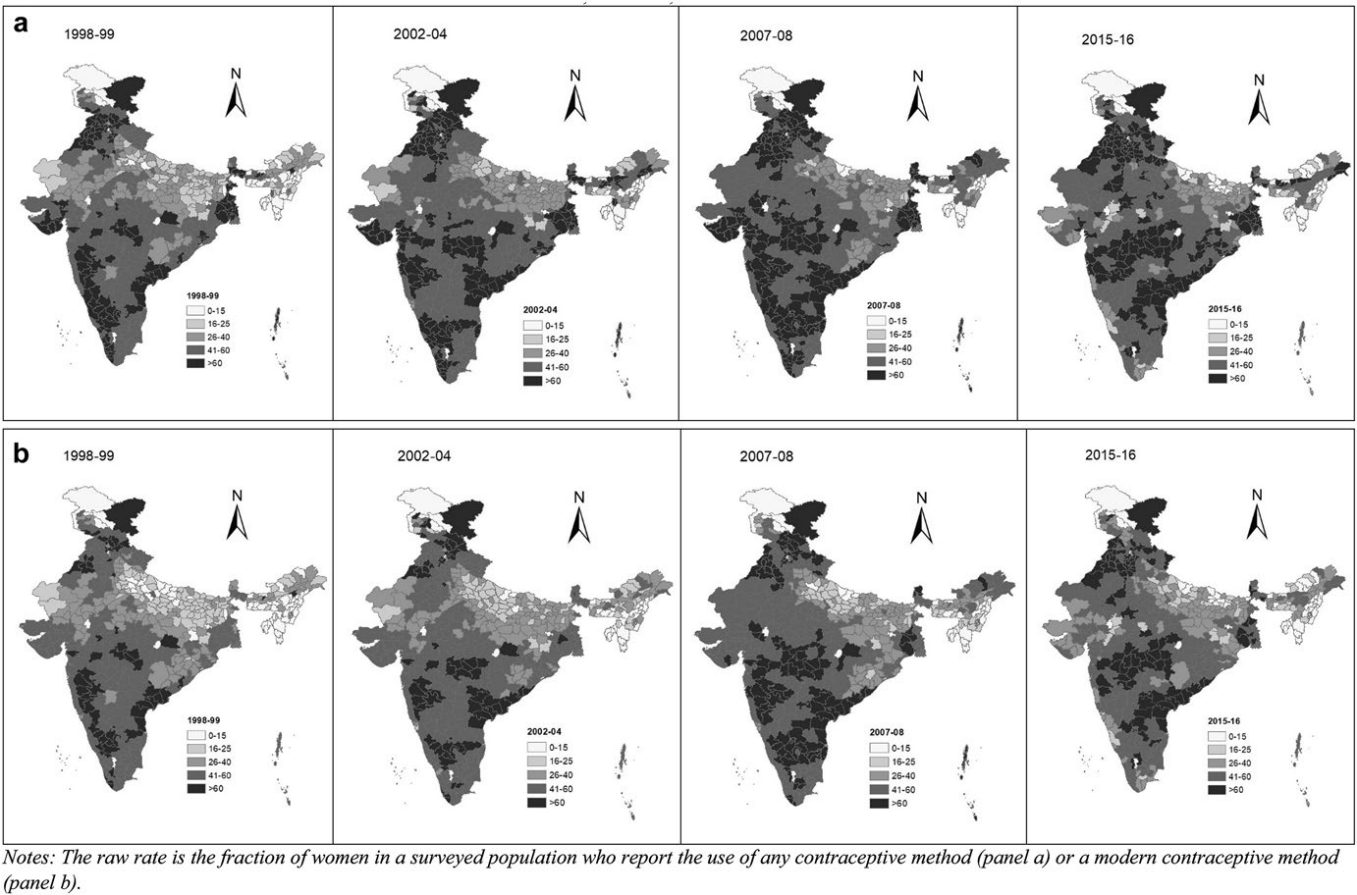
**a** CPR in the districts of India during 1998–99, 2002–04, 2007–08, 2015–16, **b**: mCPR in the districts of India during 1998–99, 2002–04, 2007–08, 2015–16

Our analysis of spatial models begins with the construction of chloropleth maps for CPR and mCPR for the four time periods. Prevalence is grouped into 5 categories: < 15%, 15–25%, 25–40%, 40–60%, > 60%. Figure 2a and b present the CPR and mCPR for 1998–99, 2002–04, 2007–08 and 2015–16. The maps highlight some interesting spatial patterns of the contraceptive prevalence in India. During 1998–99, districts with high CPRs i.e. CPRs close to 60%, were mainly in the southern states of Andhra Pradesh, Karnataka, Kerala and Tamil Nadu. In the subsequent survey years, more southern districts entered this group. Moreover, some northern districts, in the states of Jammu & Kashmir, Himachal Pradesh and Punjab also entered the group. The progress in the northeastern states is particularly striking. Most of these districts had a CPR of less than 25% in 1998–99 but increased to more than 40% in the later periods.

This suggests that there has been some localized convergence in India, i.e. some districts that had low CPRs twenty years ago did experience considerable growth in CPRs. With the exception of the northeastern states, these were largely close to districts that were already performing very well and in regions which had already made considerable strides. In contrast to these districts, there are also many districts in India where the CPR has changed very little over the sample period. These districts, mainly in Uttar Pradesh, Bihar, Rajasthan and coastal parts of Gujarat, had contraceptive prevalence rates less than 25% in 1998–99 and they continue to be in this group even after 20 years. These districts tend to lie in regions where CPRs had also been historically low. This suggests the presence of spatial autocorrelation in the data.

### Measures of Spatial Autocorrelation: Moran’s I

We first use the univariate Moran’s I Index to measure the extent of spatial autocorrelation. As mentioned earlier, we use the queen’s weights available in GeoDa. A close look at the weights suggests that each district had an average of 3.5 neighbors (and a median of 4.00).

Estimated values of Moran’s I for all explanatory variables in our analysis are presented in Table 1, along with other summary statistics. The estimated Moran’s I values for the key dependent variables are presented in Table 2. As discussed earlier, these values are expected to fall between the expected range of − 1 (perfect dispersion) to + 1 (perfect correlation). These estimates suggest high degree of correlation in the contraceptive prevalence rates (both CPR & mCPR). For Table 2, the estimates are accompanied by positive z-statistics and statistically significant p-values for all estimates. Although there is a small reduction in the observed coefficient value over the sample period, the estimate for both measures of contraceptive prevalence remains positive and significant. It is important to note that Moran’s I is an inferential statistic that can only be interpreted in the context of the null hypothesis, which states that the CPR and mCPRs are randomly distributed across the districts of India, with spatial correlations arising from random chance. The positive z-values and statistically significant p-values allow us to reject the null hypothesis. The spatial distribution of observed values are thus more spatially clustered than would be expected if underlying spatial processes were random.

**Table 2:** Univariate Moran’s I for Spatial autocorrelation in CPR and mCPR in districts of India during 1998–99, 2002–04, 2007–08, 2015–16

Year	CPR	mCPR
1998–99	0.75***	0.79***
2002–04	0.68***	0.76***
2007–08	0.73***	0.80***
2015–16	0.67***	0.73***

We denote significance at the 1% level with ***. Significance was calculated using the permutation methods. We use 999 permutations, a pseudo p-value of 0.001 and our own specified seed. The z-statistics and sample histograms were analyzed for significance

Next, we estimate bivariate Moran’s I values for our dependent variables. We present estimates for three sets of correlates: district averages of female literacy, female age at marriage and utilization of ANC services. These estimates are presented in Table 3. All the reported correlations are positive, but we note that the correlations of CPR & mCPR with female literacy and ANC utilization are greater in magnitude than the age at marriage, in all the time periods. The weaker relationship between contraceptive prevalence and the age at marriage is consistent with existing literature on India, which emphasizes that the age of marriage, a variable that is closely related to the type of marriage and household structure, has changed slower over this time period than female education or access to health services, which are more closely tied to policy in not just India, but also other parts of South Asia (Ghimire & Axinn, 2013; Moore et al., 2009). All these results are significant with the parameters specified earlier.

**Table 3:** Bivariate Moran’s I for spatial association of CPR and mCPR with selected socioeconomic development indicators in districts of India during 1998–99, 2002–04, 2007–08, 2015–16

	1998–99	2002–04	2007–08	2015–16
CPR				
Female literacy	0.41*** (12.85)	0.41*** (13.07)	0.39*** (12.75)	0.24*** (8.36)
Mean age at marriage	0.17*** (6.04)	0.22*** (7.87)	0.28*** (9.88)	0.14*** (5.06)
ANC utilization	0.59*** (18.23)	0.58*** (17.62)	0.45*** (14.83)	0.47*** (14.70)
mCPR				
Female literacy	0.34*** (11.29)	0.31*** (10.71)	0.29*** (10.23)	0.27*** (9.67)
Mean age at marriage	0.14*** (4.89)	0.12*** (4.51)	0.17*** (6.17)	0.12*** (4.37)
ANC utilization	0.58*** (18.04)	0.63*** (19.07)	0.41*** (13.77)	0.48*** (15.16)

*N* = 490 districts; We denote significance at the 1% level with

***. Significance was calculated using the permutation methods.

We use 999 permutations, a pseudo p-value of 0.001 and our own specified seed. The z-statistics and sample histograms were analyzed for significance

This overall evidence of geographical clustering is in line with the other recent research. Estimates of Moran’s I for fertility rates, constructed with census data between 1960 and 1990, produced an estimate of approximately 0.5 for districts whose headquarters are separated by 50 km or less (Guilmoto & Rajan, 2001). Estimates of Moran’s I for indicators of malnutrition also follow a similar pattern. Recent analysis suggests that estimates of this statistic for children’s stunting, wasting and incidence of being underweight are 0.65, 0.51 and 0.74 respectively (Khan & Mohanty, 2018). Other methods of quantifying clustering have also resulted in similar conclusions. In recent work, Striessnig and Bora (2019) use principal component analysis and cluster analysis on the NFHS round 4 survey data. They too find strong clustering of child health, nutrition and development indicators across districts. Rural districts in central and northern India show consistently worse outcomes for children than the districts of the southern states. There is also clustering in the northeastern states: some contiguous districts offer more favorable living conditions for children’s height and weight indicators than the rest of the region (Striessnig & Bora, 2019).

#### Bivariate Spatial Association: Local Indicators of Spatial Association (LISA) Statistics

**Fig. 3:**
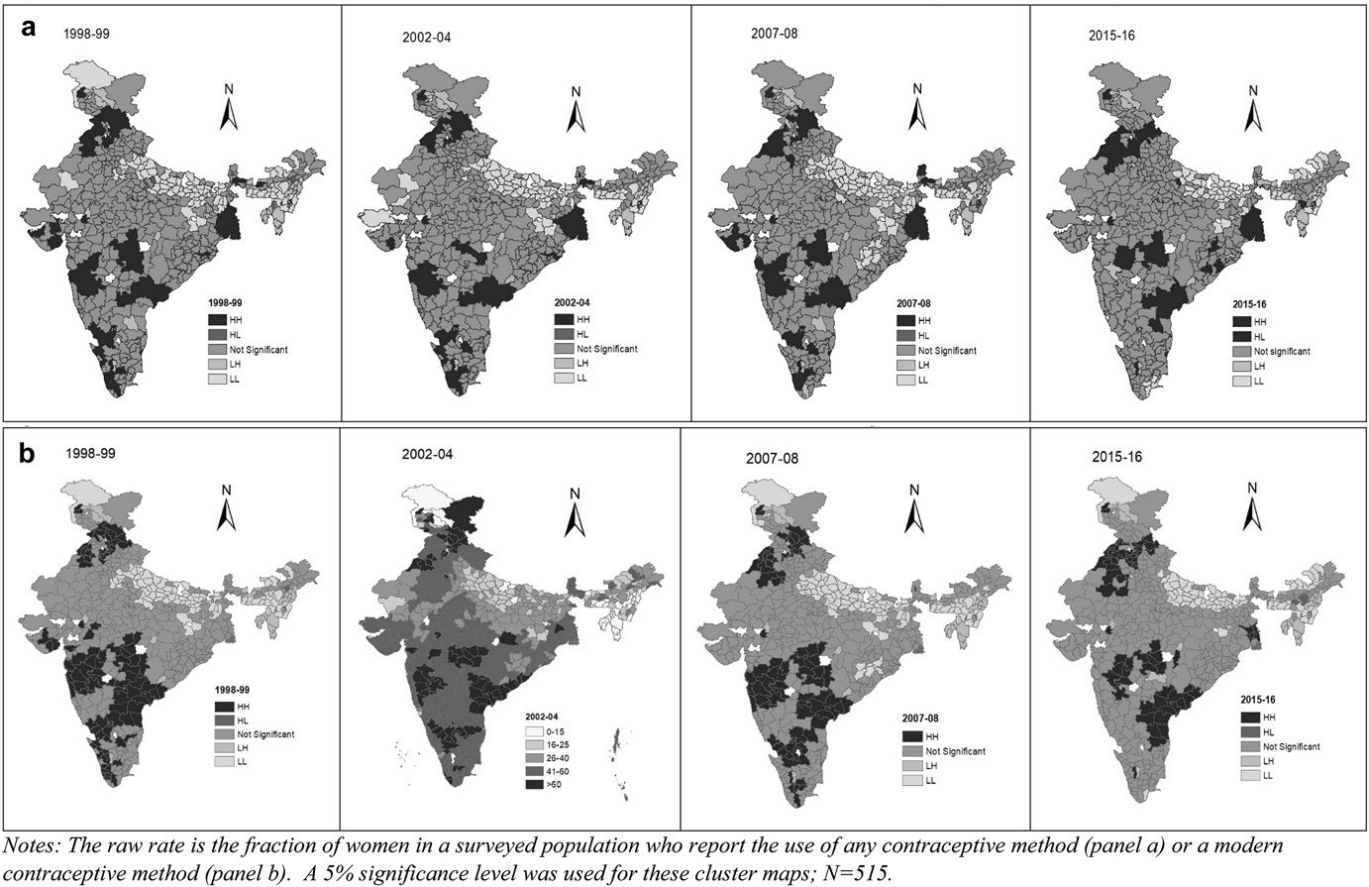
a, Univariate LISA for spatial clustering of CPR across districts of India during 1998–99, 2002–04, 2007–08, 2015–16, b: Univariate LISA for spatial clustering of mCPR across districts of India during 1998–99, 2002–04, 2007–08, 2015–16

Univariate LISA maps of CPR and mCPR are shown in Figs. 3a and b respectively. These maps show high-high clustering (dark shading), low-low clustering (light shading), and the spatial outliers (low–high and high-low clusters in medium shading). The high-high clusters are also called “hot spots” representing districts with high CPR/mCPR surrounded by districts with similar high CPR/mCPR. The low-low clusters are termed as “cold spots” and characterised by low CPR/mCPR districts surrounded by low CPR/mCPR districts. The strongly colored regions are therefore those that contribute significantly to a positive global spatial autocorrelation outcome, while paler colors contribute significantly to a negative autocorrelation outcome. The districts marked as “not significant” are those which are surrounded by districts with different patterns of CPR and mCPR.

Figure 3a, which presents the maps for CPR, indicates the presence of “hot spots” in the states of Andhra Pradesh, Karnataka, Kerala in South, Himachal Pradesh and Punjab in North, and West Bengal in the East. The results also identify “cold spots” in Uttar Pradesh, Bihar, Jharkhand and in some parts of the North-eastern states. Similar pattern is identified for clustering of mCPR. Himachal Pradesh, Maharashtra and Andhra Pradesh are the three states with “hot-spots” for mCPR over time. “Cold-spots” for mCPR are identified in Uttar Pradesh, Bihar, Jharkhand and North-Eastern states. Figure 3b, which contains maps for the mCPR, reflects same observations. It is interesting to note that the South contains fewer hotspots at the end of the time-period of study.

**Fig. 4:**
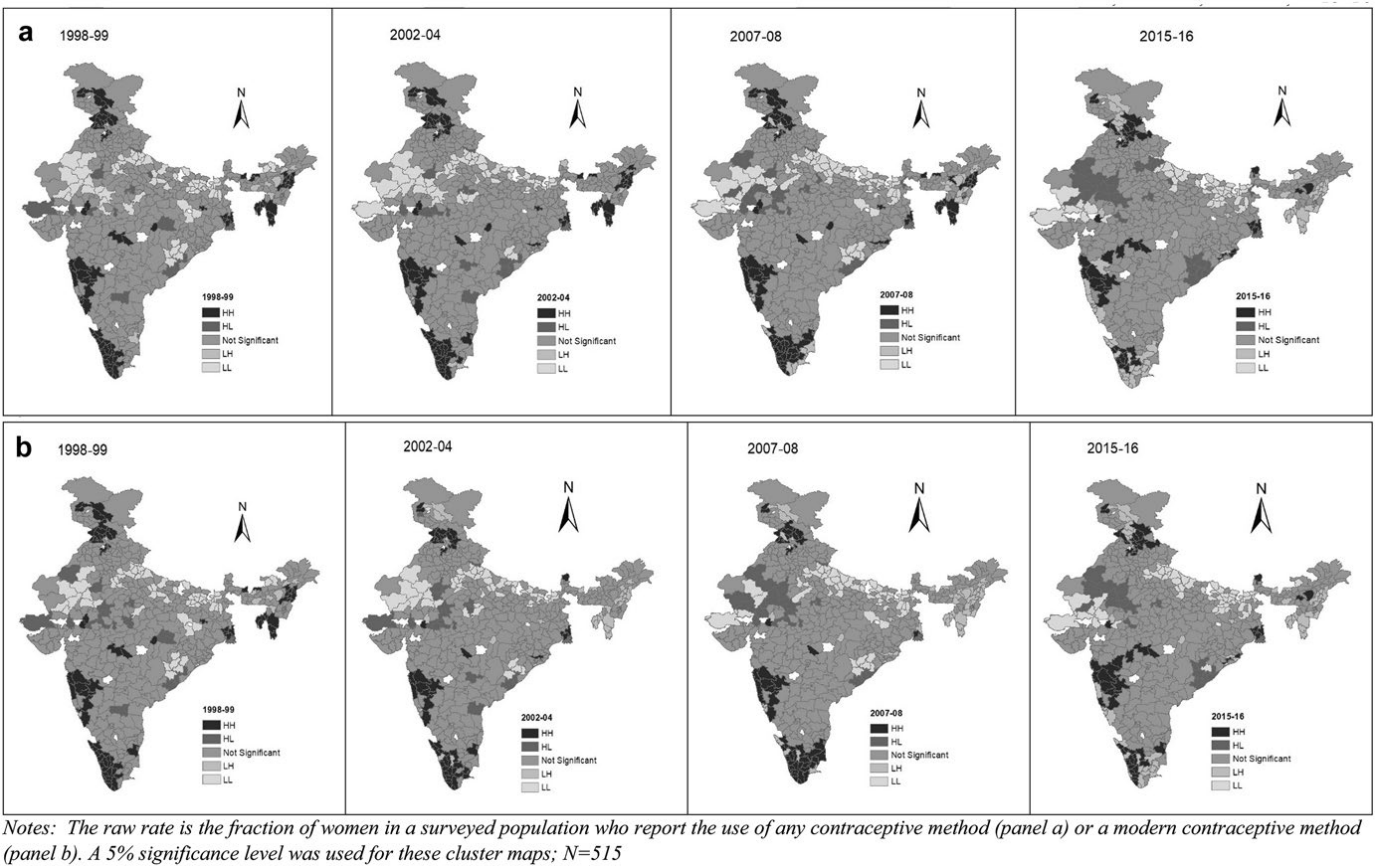
a, Bivariate LISA maps for spatial clustering of CPR with female literacy across districts of India during 1998–99, 2002–04, 2007–08, 2015–16, b: Bivariate LISA maps for spatial clustering of mCPR with female literacy across districts of India during 1998–99, 2002–04, 2007–08, 2015–16

**Fig. 5:**
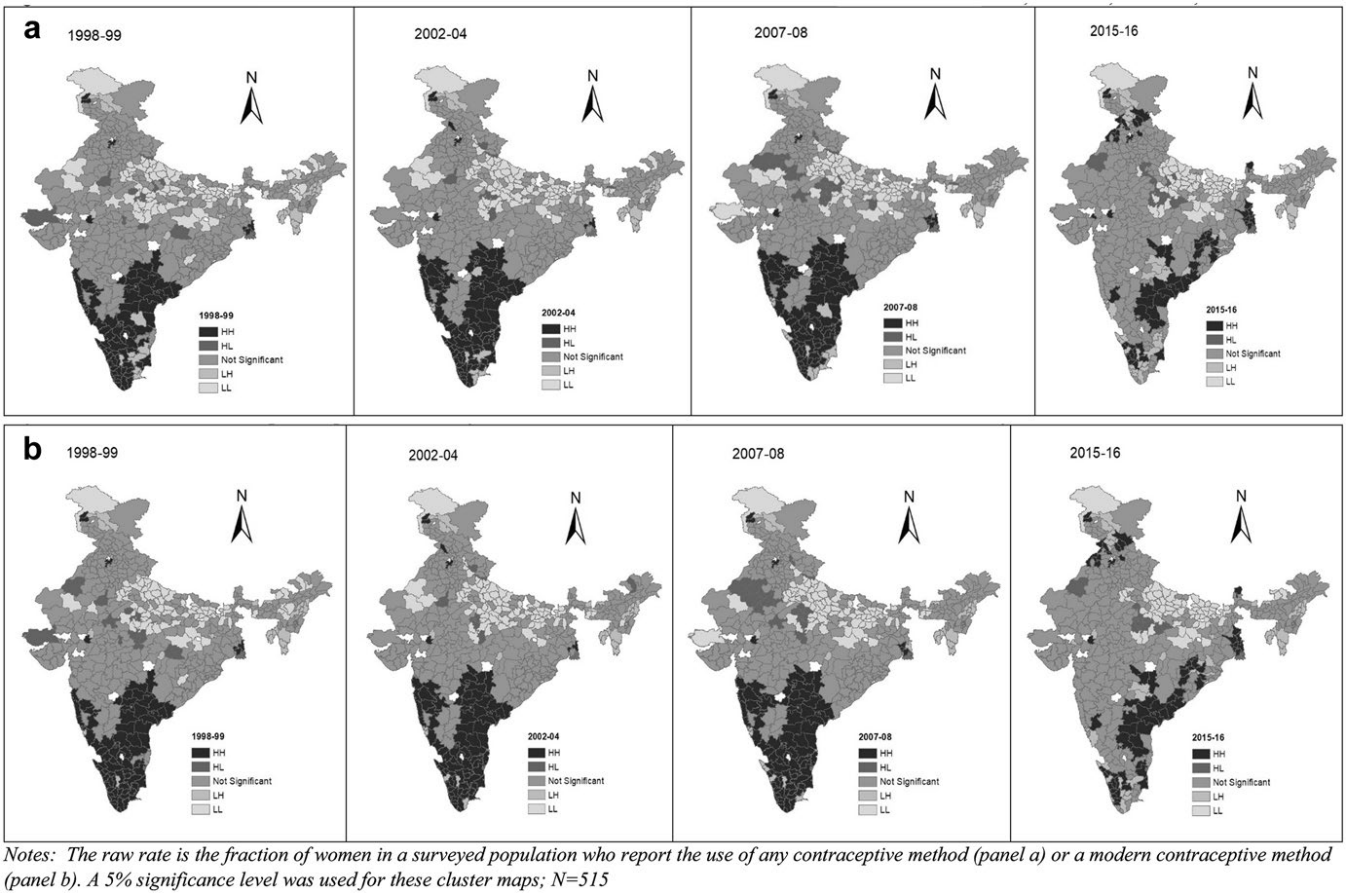
a Bivariate LISA maps for spatial clustering of CPR with ANC utilization across districts during 1998–99, 2002–04, 2007–08, 2015–16, b: Bivariate LISA maps for spatial clustering of mCPR with ANC utilization visits across districts during 1998–99, 2002–04, 2007–08, 2015–16

Next, we turn to the bivariate LISA models, which measure the local correlation between a variable and weighted average of another variable in the area around it. We explore whether the geographic clustering of contraceptive use is also correlated with one of the three variables we examined earlier: district-level average female literacy, and utilization of ANC services.^13^ Figures 4a and b present the bivariate LISA maps for spatial clustering of the CPR and the mCPR with female literacy across the districts for all survey rounds respectively. Figures 5a and b present the results of the same analysis with the utilization of ANC services. Again, we use a significance level of 5%. In results not shown here we also check the robustness of these results using significance levels of 1% and 0.1% and the Bonferroni bound procedure.^14^

The results presented in Fig. 4a suggest that several regions had high contraceptive use as well as high levels of female literacy. However, these were mainly concentrated in southern states. Some districts in the north—mainly in the state of Himachal Pradesh—also fit this pattern. The results also show that there are also many districts that had low contraceptive use as well as low levels of female literacy and these are mainly concentrated in northern states such as Rajasthan, Uttar Pradesh and Bihar. It is also interesting to note that in the last two survey rounds, there are some “high-low” districts, i.e. districts with above average contraceptive prevalence, surrounded by districts with below average values of female literacy. These are mainly concentrated in the northwest state of Rajasthan.

Similar observations can also be made of ANC utilization for both the CPR and mCPR. In Figs. 5a and b, we see that in the first round of the survey, the “high-high” clusters were almost entirely in the southern states, but over time, we see more such clusters emerge in Himachal Pradesh and even the eastern states. It is also striking that “low-low” clusters persist in the plain states, which correspond to the states of Uttar Pradesh, Jharkhand, Bihar and Chhatisgarh.

These patterns are consistent with recent findings in the literature on India’s spatial heterogeneity. Several recent papers, many using the NFHS-4, also highlight stark intra-state and inter-regional disparities with correlated measures of child health, undernutrition, and female illiteracy with other types of disadvantage such as low female education (Khan & Mohanty, 2018; Singh et al., 2011). The persistence of disparities in some regions undermines the convergence of demographic processes, i.e. not all underdeveloped clusters appear to catchup with their developed counterparts.

A caveat to these findings is in order. When we use a significance level of 1% and 0.1%, we find that all the “high-high” and “low-low” hotspots remain, but the “high-low” and “low–high” clusters disappear. The impositions of the Bonferroni bounds result in *only* the “high-high” and “low-low” clusters being significant—this is seen in all rounds of the surveys. To conserve space, we present the cluster-maps only for the case of the 5% significance with the emphasis that they must be interpreted cautiously—many such clusters could indeed be “false positives” (Anselin, 2019). We simply point out that these are possibly interesting locations in the context of contraceptive prevalence and its correlates in India.

Despite this caveat, the contrast between univariate and bivariate models provides insights into the drivers of contraceptive prevalence in India. Consider the case of the mCPR for the year 1998–99. Figure 3b, which presents the univariate mCPR, contains 102 districts in high-high category for this year. Figure 4b, which presents the bivariate plot, contains 57 districts in this category. Further analysis of the districts shows that 31 districts are common in both univariate and bivariate LISA. We find similar results for the low-low category. Figure 3b has 89 districts in the low-low category while Fig. 4b has 57 districts in this category. Here too, we find that 31 districts are common in univariate and bivariate LISA.

These patterns persist in subsequent survey rounds. In the univariate LISA plots for 2002–04, 86 districts (Fig. 3b) fall in the high-high category. In the bivariate LISA (Fig. 5b), 57 districts fall in this category. Out of these districts 21 are common in both univariate and bivariate LISA, and most of these common districts are from Kerala, Tamil Nadu and Maharashtra. The results also show that 83 districts (Fig. 3b) and 49 districts (Fig. 4b) fall in the low-low cluster in the univariate and bivariate plots respectively. Here, 25 districts are common to both and these fall in Uttar Pradesh and Bihar.

In the next survey round (2007–08), 84 and 55 districts fall in the high-high cluster from univariate and bivariate LISA respectively with 23 districts in common. In this case, the common districts fall in Himachal Pradesh, Jammu & Kashmir, Maharashtra and Tamil Nadu. The emergence of Himachal Pradesh in housing high-high districts in both the univariate and bivariate plots is interesting, for it is known to have made considerable strides in promoting female education and investments in health (Drèze & Sen, 2002). Finally, in 2015–16 also 20 districts are common out of the univariate (74 districts) and bivariate (58 districts). The common districts are from Haryana, Himachal Pradesh, Maharashtra, Punjab and West Bengal. Again, the emergence of new states in the list of areas where high-high clusters are located is an interesting development.

Given the caveats discussed earlier about the robustness of these findings, we believe that it is simply sufficient to conclude that contraceptive utilization in India has strong spatial variation at the district level. Variables such as female literacy and investments in health infrastructure have strong, but not perfect associations with the contraceptive prevalence rate. In other words, India has a significant number of districts where contraception adoption rates are high (low), and these are not always the same places where investments in female education or health services have occurred.

## Regression Models

So far, we have found considerable evidence of localised clustering of contraceptive use in India, with some evidence that this clustering is correlated with variables such as female literacy and access to healthcare. The final step of our analysis is to examine the correlates of contraceptive prevalence in a multivariable framework.

Given the considerable geo-spatial clustering of both the outcome and the explanatory variables, we anticipate the need for applied spatial regression models. We begin however, with the OLS framework and incorporate all the independent variables discussed earlier in the paper—the percentage of the district that is urban, the percentage of women who are literate, the percentage of the district that belongs to disadvantaged castes and tribes, population density, the sex ratio, the district female labor force participation rate, the mean age at marriage and women’s access to health care, as measured by the fraction of women who received adequate ante-natal care for births in the preceding five years.

The results from regressions, obtained via the package of spatial tools in STATA 16 as well as GeoDa, are summarised in Table 4. We immediately note that there is strong evidence of spatial clustering. The spatial lag regressions and spatial error models each highlight a considerable level of clustering with p-values of the tests of spatial correlation being 0. Diagnostics of the OLS regressions are presented in Appendix Tables 2 and 3—these clearly indicate the presence of spatial correlation and the use of both the spatial lag and spatial error models.^15^

**Table 4:** Results of OLS and spatial regressions for determinants of CPR across districts of India during 1998–99, 2002–04, 2007–08, 2015–16

	1998–99 (N = 490)			2002–04 (N = 490)			2007–08 (N = 490)			2015–16 (N = 490)		
	OLS	Spatial lag	Spatial error	OLS	Spatial lag	Spatial error	OLS	Spatial lag	Spatial error	OLS	Spatial lag	Spatial error
Female literacy	0.357**	0.333***	0.353***	0.262*	0.240***	0.299***	0.171	0.174***	0.211***	− 0.019	0.001	0.005
	(0.117)	(0.046)	(0.045)	(0.101)	(0.046)	(0.047)	(0.101)	(0.044)	(0.043)	(0.097)	(0.051)	(0.054)
Scheduled caste/tribe	− 0.180*	− 0.111***	− 0.129***	− 0.162*	− 0.125***	− 0.199***	− 0.085	− 0.044	− 0.078**	− 0.117	− 0.054	− 0.097**
	(0.075)	(0.030)	(0.027)	(0.073)	(0.030)	(0.029)	(0.075)	(0.027)	(0.027)	(0.071)	(0.029)	(0.031)
Muslim	0.001	0.082*	− 0.040	− 0.031	0.019	− 0.078	− 0.109	− 0.054	− 0.117***	− 0.111	− 0.005	− 0.072
	(0.102)	(0.038)	(0.040)	(0.082)	(0.037)	(0.041)	(0.066)	(0.034)	(0.034)	(0.069)	(0.037)	(0.041)
Mean age at marriage	− 2.448	− 1.629**	− 2.100***	− 1.376	− 0.863	− 1.658**	− 0.661	− 0.463	− 1.325**	− 2.384	− 1.780***	− 2.713***
	(1.626)	(0.501)	(0.596)	(1.294)	(0.463)	(0.585)	(1.033)	(0.403)	(0.462)	(1.289)	(0.479)	(0.555)
Women received3 + ANC	0.252***	0.239***	0.204***	0.313***	0.322***	0.291***	0.241***	0.275***	0.228***	0.612***	0.594***	0.553***
	(0.062)	(0.031)	(0.035)	(0.065)	(0.028)	(0.035)	(0.047)	(0.029)	(0.035)	(0.111)	(0.035)	(0.041)
Urban	− 0.026	− 0.028	− 0.070*	− 0.120	− 0.135**	− 0.103**	0.037	0.029	− 0.020	− 0.069	− 0.059	− 0.052
	(0.080)	(0.038)	(0.035)	(0.068)	(0.044)	(0.040)	(0.065)	(0.034)	(0.030)	(0.049)	(0.032)	(0.033)
Population Density	− 0.036	− 0.032	− 0.015	0.003	0.007	− 0.001	− 0.041	− 0.042	− 0.011	0.008	0.008	0.018
	(0.039)	(0.028)	(0.024)	(0.034)	(0.028)	(0.024)	(0.040)	(0.025)	(0.021)	(0.032)	(0.027)	(0.025)
Sex ratio	− 0.053*	− 0.039***	− 0.021*	− 0.055**	− 0.044***	− 0.035***	− 0.064***	− 0.059***	− 0.032***	− 0.070**	− 0.049***	− 0.022
	(0.020)	(0.010)	(0.011)	(0.017)	(0.009)	(0.010)	(0.013)	(0.009)	(0.009)	(0.024)	(0.010)	(0.011)
Female labor force part	0.141	0.157*	− 0.069	0.068	0.085	0.020	0.156	0.216**	0.069	− 0.136	0.006	− 0.075
	(0.170)	(0.069)	(0.074)	(0.182)	(0.067)	(0.074)	(0.206)	(0.072)	(0.070)	(0.207)	(0.077)	(0.084)
Average household size	− 4.845*	− 5.925***	− 3.363**	− 2.895	− 3.756***	− 0.015	− 3.605	− 3.588***	− 3.440***	− 0.138	− 1.932*	− 1.359
	(1.959)	(0.901)	(1.039)	(1.764)	(0.890)	(1.059)	(1.981)	(0.870)	(0.907)	(1.919)	(0.932)	(1.078)
Constant	141.036***	92.407***	105.260***	119.523***	85.432***	92.172***	120.163***	88.056***	103.001***	132.469***	70.519***	99.178***
	(32.238)	(14.658)	(14.245)	(27.492)	(13.547)	(13.966)	(23.988)	(12.724)	(11.905)	(29.978)	(13.875)	(14.405)
ρ		0.268***			0.202***			0.264***			0.399***	
		(0.072)			(0.026)			(0.028)			(0.032)	
Λ			0.7270***			0.538***			0.699***			0.6520***
			(0.027)			(0.039)			(0.088)			(0.032)
R2/Pseudo- R2	0.580	0.764	0.546	0.612	0.642	0.590	0.428	0.538	0.507	0.489	0.545	0.474
Log-likelihood	− 578.21	− 445.241	− 439.2166	− 1812.47	− 1783.72	− 1759.13	− 1859.62	− 1814.65	− 1728.29	− 2022.94	− 1951.61	− 1911.30
Akaike info criterion	1178.07	914.482	900.433	3646.94	3591.44	3540.28	3741.24	3653.29	3478.57	4067.89	3927.23	3844.62
Likelihood Ratio Test		265.7312	277.7803		57.5012	106.65		89.9482	262.6667		142.6609	223.2708

We denote significance at the 1%, 5% and 1% level with ***, ** and * respectively

The significant values of ρ (spatial lag term) and λ (spatial error term) in both Table 4 and 5 confirm the presence of significant spatial correlation in our data. The increased value of ρ over time in the spatial error models in both tables suggests that spatial clustering may be strengthening over time. This trend is not as apparent for λ, which also started out at a higher value in almost all the regressions. While it is tempting to interpret this as evidence of spatial dependence in observables, and a diminishing role of spatial dependence in unobservables, the inherent limitations of both the spatial lag and spatial error model make that difficult (Gibbons & Overman, 2012).

**Table 5:** Results of OLS and spatial regressions for the determinants of mCPR across districts of India during 1998–99, 2002–04, 2007–08, 2015–16

	1998–99 (N = 490)			2002–04 (N = 490)			2007–08 (N = 490)			2015–16 (N = 490)		
	OLS	Spatial lag	Spatial error	OLS	Spatial lag	Spatial error	OLS	Spatial lag	Spatial error	OLS	Spatial lag	Spatial error
Female literacy	0.215	0.200***	0.261***	0.053	0.041	0.147***	0.085	0.103*	0.186***	0.019	0.198***	0.242***
	(0.106)	(0.043)	(0.039)	(0.091)	(0.040)	(0.040)	(0.099)	(0.045)	(0.042)	(0.083)	(0.054)	(0.064)
Scheduled caste/tribe	− 0.211**	− 0.129***	− 0.126***	− 0.145*	− 0.085**	− 0.088***	− 0.104	− 0.032	− 0.050	− 0.132*	− 0.069*	− 0.093**
	(0.061)	(0.028)	(0.024)	(0.059)	(0.026)	(0.025)	(0.063)	(0.028)	(0.026)	(0.049)	(0.027)	(0.028)
Muslim	− 0.033	0.050	− 0.086*	− 0.136*	− 0.066*	− 0.156***	− 0.231**	− 0.136***	− 0.200***	− 0.156*	− 0.062	− 0.047
	(0.106)	(0.035)	(0.035)	(0.066)	(0.033)	(0.034)	(0.068)	(0.035)	(0.034)	(0.070)	(0.034)	(0.046)
Mean age at marriage	− 1.864	− 1.009*	− 1.834***	− 1.667	− 0.916*	− 1.573**	− 1.289	− 0.894*	− 1.525***	− 2.843*	− 2.220***	− 2.721***
	(1.595)	(0.465)	(0.511)	(1.266)	(0.406)	(0.493)	(1.331)	(0.416)	(0.459)	(1.224)	(0.448)	(0.520)
Women received3 + ANC	0.295***	0.266***	0.179***	0.449***	0.431***	0.330***	0.320***	0.346***	0.215***	0.546***	0.517***	0.424***
	(0.058)	(0.029)	(0.031)	(0.057)	(0.025)	(0.031)	(0.061)	(0.030)	(0.035)	(0.083)	(0.033)	(0.038)
Urban	0.035	0.019	− 0.037	− 0.024	− 0.054	− 0.039	0.135*	0.107**	0.051	0.019	0.014	− 0.001
	(0.078)	(0.036)	(0.031)	(0.059)	(0.039)	(0.034)	(0.066)	(0.035)	(0.030)	(0.044)	(0.030)	(0.030)
Population Density	− 0.069	− 0.058*	− 0.024	− 0.025	− 0.015	− 0.013	− 0.080*	− 0.074**	− 0.032	− 0.011	− 0.009	0.003
	(0.038)	(0.026)	(0.021)	(0.033)	(0.024)	(0.021)	(0.037)	(0.025)	(0.021)	(0.030)	(0.025)	(0.022)
Sex ratio	− 0.066**	− 0.047***	− 0.029**	− 0.068***	− 0.052***	− 0.040***	− 0.072***	− 0.063***	− 0.039***	− 0.069**	− 0.052***	− 0.033**
	(0.018)	(0.009)	(0.009)	(0.013)	(0.008)	(0.009)	(0.015)	(0.009)	(0.009)	(0.021)	(0.010)	(0.010)
Female labor force part	0.451**	0.424***	0.134*	0.302*	0.294***	0.169**	0.370	0.439***	0.270***	0.126	0.219**	0.096
	(0.145)	(0.065)	(0.065)	(0.139)	(0.059)	(0.062)	(0.238)	(0.074)	(0.070)	(0.213)	(0.072)	(0.077)
Average household size	− 4.314*	− 5.466***	− 3.500***	− 1.757	− 2.969***	− 1.147	− 3.774	− 3.561***	− 2.742**	− 1.678	− 2.870***	− 0.618
	(1.820)	(0.838)	(0.892)	(1.649)	(0.780)	(0.910)	(2.474)	(0.896)	(0.901)	(2.199)	(0.863)	(1.019)
Constant	128.971***	77.490***	101.406***	117.389***	72.624***	91.348***	126.888***	75.937***	97.396***	133.875***	79.356***	97.357***
	(28.390)	(13.522)	(12.393)	(22.715)	(11.749)	(11.566)	(27.202)	(13.023)	(11.828)	(30.415)	(13.019)	(13.398)
ρ		0.252***										
(0.032)			0.271***									
(0.029)			0.348***									
(0.029)			0.421***									
(0.032)												
λ			0.730***			0.638***			0.788***			0.697***
			(0.027)			(0.033)			(0.022)			(0.029)
R^2^/Pseudo- R^2^	0.625	0.721	0.822	0.575	0.651	0.744	0.465	0.604	0.802	0.288	0.513	0.649
Log-likelihood	− 1832.56	− 1767.53	− 1700.77	− 1835.18	− 1792.07	− 1747.10	− 1903.25	− 1838.66	− 1722.88	− 1968.37	− 1888.91	− 1839.60
Akaike info criterion	3687.13	3559.06	3605.44	3692.37	3608.13	3516.21	3828.5	3701.32	3467.75	3958.74	3801.82	3701.21
Likelihood Ratio Test		130.0642	263.5813		86.2373	176.1613		129.1793	360.741		158.92	257.53

We denote significance at the 1%, 5% and 1% level with ***, ** and * respectively

The log-likehood, AIC and Likelihood Ratio Test of spatial error dependence do not provide clear evidence in support of a specific spatial regression model. We emphasize that in the future, with population surveys that are more comparable across regions as well as time, it will be important to explore the temporal and spatial variations of these variables with panel models that include both types of spatial dependence. For now, we simply emphasize that the issue of spatial correlation is likely present in these data.

We also note that the results are quite stable across the three different regression models. For both the CPR (Table 4) and the mCPR (Table 5) we note a positive and statistically significant association in all the specifications in the first two rounds of our survey. The coefficients diminish in both magnitudes and significance after the third round. The size of the coefficients, though difficult to interpret in this framework, is also interesting. In the first three rounds in the CPR regressions (Table 4), a 1% percent increase in female literacy in a district is associated with a 0.2–0.4 percentage increase in the CPR. It is also noteworthy that the size of the effect of these determinants did not change much after controlling for spatial autocorrelation indicating robustness of the estimates.

Similarly, the percentage of SC/ST population in a district takes a significant negative coefficient in the regressions for CPR (Table 4) and mCPR (Table 5) in all four rounds, and the strongest results are seen in the spatial error models (columns 3, 6 and 9). The percentage of a district’s population that is Muslim is rarely significant in any specification—likely because other control variables are accounting for the variability. In 2007–08 however, percentage of Muslim population had significantly negative and consistent effect on the CPR in the spatial error model.

We also note that the sex ratio, defined as the number of females per 1000 males, had significant negative impact on CPR (Table 4) and mCPR (Table 5) in all the three rounds. If we interpret the sex-ratio as a measure of female disadvantage and discrimination, we see that greater levels of gender equality are associated with greater levels of contraceptive prevalence. This could largely be driven by the agency of mothers, or the preference for sons. Disentangling the causal impact is beyond the scope of the paper, but the result is consistent with the vast literature on gender inequality in India (Drèze and Sen, 2002; Dreze and Sen, 2013; Arokiasamy, 2002).

It is also interesting to note that the average population density of the district and the fraction of a district that is urban have relatively weak associations in all the specifications, including the spatial regressions in both Tables 4 and 5.

The strongest level of statistical significance is seen in the measure of health services—the fraction of women in a district who have received 3 + ANC visits in the event of any pregnancy in the five years before the surveys. We note that this variable is positive and statistically significant at the 1% level in all specifications in all rounds for both the CPR (Table 4) and the mCPR (Table 5). The magnitude of the coefficient has almost doubled between Round 1 and Round 4. In Table 4 we see that the coefficient in the spatial error model stood at 0.239 (Spatial Lag model, 1998–99 Round) and increased to 0.594 by 2015–16 (Spatial Lag model). Similarly, in Table 5 the coefficient in the spatial error model stood at 0.266 in Round 1 and this coefficient increased to 0.517 by Round 4. Clearly, access to health services is one of the biggest and most robust correlates of the adoption of contraception in the past twenty years.

In summary, we find evidence for spatial correlation as well as broad relationships between these variables that is in line with previous literature: CPR and mCPR is positively associated with female education, negatively with SC/ST, Muslim population, household size and population sex-ratio. It is noteworthy that the contraceptive prevalence rate is correlated with *both* demand-side variables such as female education and the age at marriage *and* also supply-side variables such as the availability and quality of health care infrastructure. This suggests that the clustering of CPRs and mCPRs is likely driven by the clustering of socio-economic development as well as investments in health infrastructure in certain states and districts of the country.

## Summary and Conclusion

This paper contributes to the literature on district-level variations and spatial clustering of contraceptive use in India. We draw on four national population-based household surveys: District-Level Household Survey (DLHS, 1998–99, 2002–04 & 2007–08) and National Family Health Survey-4 (NFHS, 2015–16). We aggregate these surveys at the district level, with some adjustments to make the four surveys comparable. Comparisons of averages and ranges of CPR and mCPR values at district-level across the four cross section suggested considerable variation across the districts of India. In all rounds of data, the minimum observed prevalence of the CPR and mCPR was below 7 percent, and the maximum was 80—90 percent. This range is considerably higher than what we observe at the state level.

Next, we examined spatial clustering of CPR and mCPR at district level across four time periods using spatial models. Univariate Moran’s I consistently reveal evidence of significant clustering in the spatial distribution of contraceptive prevalence rates across districts. Bivariate estimates suggest that the prevalence of the CPR and mCPR is positively correlated with other correlates such as female literacy, female age at marriage and the average rate of utilization of ANC services (a proxy for health services in a district). This suggests that not only there is spatial clustering, but this clustering is likely driven by socio-economic variables as well as investments in health-care infrastructure at the local level.

To better understand the regional variations, we rely on univariate and bivariate LISA models. Univariate models illustrate that high prevalence districts are clustered in the southern states and northern states of Himachal Pradesh. Low CPR & mCPR districts are clustered in Uttar Pradesh, Bihar, Madhya Pradesh, Jharkhand and parts of Gujarat and Rajasthan. The pattern of spatial clustering has remained remarkably persistent over the study period. Bivariate models suggest that the spatial pattern of contraceptive prevalence is associated with the spatial pattern of socioeconomic development. There are many districts where high contraceptive prevalence is explained by female literacy and the provision of ANC services. In 1998–99, these were largely clustered in Southern Indian states. Over the subsequent three rounds however, the number of “hot spots” where high contraceptive prevalence is explained by these variables has grown over time in states such as Haryana, Himachal Pradesh, Maharashtra, Punjab and West Bengal.

The final step of our analysis is to examine the determinants of contraceptive prevalence using multivariate regression models with spatial lags and errors. On the whole, these results suggest that female literacy, caste, religion and access to ANC all had a consistent and significant association with contraceptive use across all four rounds of the survey. These relationships are remarkably robust over time and to the consideration of spatial correlations. The most important determinant of the spatial variation of contraceptive use however, is our measure of health care investment (access to ANC services by women in a district). This suggests that local investments in women’s human capital as well as health-care infrastructureare associated with the adoption of contraception.

These findings are important considering that many large-scale policies have deliberately focused on reducing health inequalities in India and improving women’s access to the health care system. The National Rural Health Mission (NRHM) for example, clearly identified high and low-performing states and invested considerably in the latter group. Our analysis shows that low contraceptive clusters continue to be in these states, even as India displays a convergence of contraceptive prevalence. In the light of FP2020 goal, the findings from this study have important implications. Targeting both contraception and investment in women’s human capital to specific clusters of districts within states could be a promising way forward.

## Appendix

See Tables 6, 7 and 8.

**Table 6:** Recoding of districts to link the four surveys

State	Number of districts recoded in DLHS 2	Number of additional districts recorded in DLHS 3	Number of additional districts recorded in NFHS 4	Number of districts dropped
Arunachal Pradesh	0	3	0	0
Assam	0	3	0	1
Bihar	11	4	0	1 (DLHS 2)
Gujarat	0	3	0	0
Haryana	0	3	0	0
Jammu & Kashmir	0	0	0	1
Karnataka	6	0	0	1
Madhya Pradesh	0	14	0	1
Maharashtra	6	0	0	0
Manipur	2	0	0	0
Punjab	0	2	0	1
Rajasthan	2	0	0	0
Tamil Nadu	9	1	0	0
Uttar Pradesh	16	0	0	0
West Bengal	2	0	0	0
Union Territories	8	0	0	0

**Table 7:** Regression Diagnostics, OLS Regression, CPR

	1998–99 (N = 490)	2002–04 (N = 490)	2007–08 (N = 490)	2015–16 (N = 490)
Moran’s I (error)	15.8916***	10.2591***	15.9004***	15.1704***
Lagrange Multiplier (lag)	265.0807***	59.4889***	94.7861***	153.0432***
Robust LM (lag)	40.3546***	10.3708***	2.4441	8.4020***
Lagrange Multiplier (error)	236.2939***	96.4384***	237.1496***	215.5190***
Robust LM (error)	11.5679***	47.3204***	144.807***	70.8777***
Lagrange Multiplier (SARMA)	276.6486***	106.8093***	239.5937***	223.9209***

We denote significance at the 1%, 5% and 1% level with ***, ** and * respectively

**Table 8:** Regression Diagnostics, OLS Regression, mCPR

	1998–99 (N = 490)	2002–04 (N = 490)	2007–08 (N = 490)	2015–16 (N = 490)
Moran’s I (error)	15.064***	13.034***	16.868***	15.530***
Lagrange Multiplier (lag)	131.822***	90.813***	133.51***	167.08***
Robust LM (lag)	14.488***	8.198***	3.1316*	10.691***
Lagrange Multiplier (error)	211.727***	157.870***	267.46***	226.09***
Robust LM (error)	94.393***	75.256***	137.08***	69.699***
Lagrange Multiplier (SARMA)	226.215***	166.069***	270.59***	236.78***

We denote significance at the 1%, 5% and 1% level with ***, ** and * respectively

## Footnotes

1 Fertility estimates are drawn from the World Bank and the United Nations Population Division. World Population Prospects: 2019 Revision. Available at https://data.worldbank.org/indicator/SP.DYN.TFRT.IN.

2 The prevalence of contraception is often referred to by demographers as a key “proximate determinant” of fertility decline since this variable is highly responsive to both demand and health policy investments at almost all stages of demographic transition (Bongaarts, 2001; Bongaarts & Potter, 2013; Bongaarts, 2015).

3 In 2012, the landmark London Summit on Family Planning mobilized governments, international agencies, civil society organizations, foundations, and the private sector to commit to dramatically expanding access to voluntary family planning. A global partnership was established—the Family Planning 2020 (FP2020)—to create global momentum on the issue of access to contraceptives and encourage collaboration, innovation, and greater accountability in family planning efforts in countries such as India. The Bill and Melinda Gates Foundation has been one of the leaders of this effort.

4 Sustainable Goal 3.7 is as follows: “By 2030, ensure universal access to sexual and reproductive health-care services, including for family planning, information and education, and the integration of reproductive health into national strategies and programmes”. Sustainable Development Goal 5.2 is as follows: “Ensure universal access to sexual and reproductive health and reproductive rights as agreed in accordance with the Programme of Action of the International Conference on Population and Development and the Beijing Platform for Action and the outcome documents of their review conferences.”.

5 At the time of writing of this paper, the Government of India also released state-level aggregate data for 22 states that were surveyed in Round 5 of the NFHS. The micro-data however, has not yet been released. We are unable to utilize this data for our analysis.

6 If a new district is created, then CPR and mCPR were calculated from the combined sample of these two districts. In this process we did not lose any information for any new/old district. So the 515 districts in our paper correspond to all the districts of India (except for two states Nagaland and Tripura) which are presented under the name of the parent district from which this district was bifurcated. The shape file (district map of India) was modified accordingly to represent 515 districts.

7 Consider the case where a large district split into two smaller districts that were unequal in size, and one district was considerably worse off on a specific indicator. After the split, the two districts would be sampled separately and the survey would be representative at the level of the district. If we now assign them to their original “parent” district, we would have an overrepresentation of the smaller (and worse off) district. This would lead to a worsening of the indicator in question.

8 The Scheduled Castes (SCs) and Scheduled Tribes (STs) are historically disadvantaged groups that comprise about 16.6% and 8.6%, of India's population respectively (Census of India, 2011). The Constitution currently recognizes 1,108 caste communities across 28 states in the SC group and 744 tribes across 22 states in the ST group. The lists of those who are included has changed over time. In each round of the survey we rely on self-reported membership at the time that the survey was conducted.

9 In its most simple form, the spatial weights matrix expresses the importance of a relationship between two locations in a dataset in binary form, with weights 1 and 0. In a dataset that describes n locations (points, areal units, network edges, etc.), the spatial weights matrix express the potential for interaction between observations at each pair (i,j) of locations.

10 The queen’ criterion for constructing weights defines neighbors as spatial units sharing a common edge or a common vertex. The alternative to this, the rook’s criterion, defines neighbors by the existence of a common edge between two spatial units. The number of neighbors according to the queen criterion however, is always at least as large as for the rook criterion.

11 Specifically, the command *spmatrix* is used on the shapefiles used earlier to create a a spectral-normalized inverse-distance spatial weighting matrix. This model evaluates clustering of an outcome variable that is not explained by the independent variables. Spatial clustering is explained with reference to the clustering of the error terms.

12 Note that these two models differ in their assumption about spillovers that come from observables (spatial lag model) versus unobservables (spatial error model). Each model assumes that spatial heterogeneity in outcomes arises from a single source, constraining the other possibilities to zero. Neither model features any assumptions about the underlying processes that generate the spatial dependence. In this case, where we seek to understand the spatial clustering of contraceptive prevalence, a complex set of factors is in play: diffusion of social norms, the spread of awareness, and the availability of the contraceptives themselves.

13 Bivariate LISA maps for the CPR and mCPR with age at marriage are omitted here because this variable yielded the lowest correlations in the analysis of the bivariate Moran’s I.

14 The standard p-value of 0.05 is likely to lead to many false positives, i.e., rejections of the null when in fact it holds. As noted by Anselin (2019) the computational permutation processes entailed in the tests of significance create this problem and there is currently no best-practice or completely satisfactory solution. The methods available in the literature and packages such as GeoDa such as the Bonferroni bounds and False Discovery Rate, allow a user to conduct an extensive sensitivity analysis. Our goal in using the traditional p-value of 0.05 is driven by the goal of understanding the interesting locations, rather than interpreting the most stringent tests.

15 We rely on Moran’s I and Lagrange Multiplier test statistics to provide insights into the need for a spatial regression model. We find p-values for the lag and error models that are below 5% in all cases and below 1% in most case. This suggests that there may be considerable spatial dependence in the data. There was no clear case for using only one of the models and so we present the results of both.
